# Effects of 12 and 24 Weeks of Interdisciplinary Interventions on Health-Related Physical Fitness, Biochemical Markers, and Level of Food Processing in Overweight or Obese Adolescents: A Longitudinal Study

**DOI:** 10.3390/ijerph21111406

**Published:** 2024-10-24

**Authors:** Lilian Rosana dos Santos Moraes, Natalia Quevedo dos Santos, Déborah Cristina de Souza Marques, Marilene Ghiraldi de Souza Marques, Marielle Priscila de Paula Silva Lalucci, Victor Augusto Santos Perli, Paulo Vitor Suto Aizava, Jordan Hernandez-Martinez, Pablo Valdés-Badilla, Braulio Henrique Magnani Branco

**Affiliations:** 1 Interdisciplinary Laboratory of Intervention in Health Promotion, Cesumar Institute of Science, Technology, and Innovation, Maringa 1610, PR, Brazil; lilian.moraes@unicesumar.edu.br (L.R.d.S.M.); natquevedo01@gmail.com (N.Q.d.S.); marques.deborah@hotmail.com (D.C.d.S.M.); marileneghiraldi@gmail.com (M.G.d.S.M.); marielle.silva@unicesumar.edu.br (M.P.d.P.S.L.); victoraperli@gmail.com (V.A.S.P.); paulo.aizava@vitru.com.br (P.V.S.A.); 2 Graduate Program in Health Promotion, Cesumar University, Maringa 1610, PR, Brazil; 3 Department of Physical Activity Sciences, Universidad de Los Lagos, Osorno 1070000, Chile; jordan.hernandez@ulagos.cl; 4 Programa de Investigación en Deporte, Sociedad y Buen Vivir, Universidad de los Lagos, Osorno 1070000, Chile; 5 Department of Physical Activity Sciences, Faculty of Educational Sciences, Universidad Católica del Maule, Talca 3530000, Chile; 6 Sports Coaching Career, School of Education, Universidad Viña del Mar, Viña del Mar 2520000, Chile

**Keywords:** obesity in adolescence, health promotion, eating habit

## Abstract

Background: The aim was to investigate the effects of 12 and 24 weeks of interdisciplinary interventions on health-related physical fitness, biochemical markers, and food processing levels in overweight or obese adolescents. Methods: Thirty-one adolescents completed 24 weeks of nutritional, psychoeducational, and physical activity interventions in addition to assessments and tests carried out before, at 12 weeks, and after 24 weeks. Results: There were increases in lean mass (*p* = 0.003) and decreases in absolute and relative fat mass (*p* < 0.001) for both sexes, as well as increases in flexibility on the right and left sides (*p* < 0.001), maximum oxygen consumption (*p* < 0.001) increased after the interventions, time spent in isometric exercises on the plank increased (*p* > 0.05), total cholesterol after 12 weeks decreased (*p* = 0.014), triglycerides were reduced (*p* = 0.002), low-density lipoproteins were reduced (*p* < 0.001), consumption of more processed foods after 24 weeks decreased (*p* < 0.001), consumption of fresh foods in grams and kilocalories increased (*p* < 0.001), and, in addition, the consumption of processed and ultra-processed foods was reduced (*p* = 0.020). Conclusions: The 24-week interventions promoted superior benefits for adolescents’ physical, nutritional, and biochemical health, although the dropout rate was high (~50%).

## 1. Introduction

Adolescent obesity has emerged as a critical public health issue globally, with significant implications for both current and future health outcomes [1,2]. Recent data suggest that approximately 250 million adolescents worldwide are either overweight or obese, a figure projected to rise to 75 million by 2025 if current trends continue [1,2]. This alarming increase is attributed mainly to the consumption of unhealthy foods, insufficient physical activity practice, as well as emotional and social aspects [3]. Obesity during adolescence is linked to a myriad of health risks, primarily due to its association with various metabolic complications and multifactorial issues, including social, cultural, biological, and economic factors [4]. Adolescents face numerous challenges in adhering to intervention programs, notably resistance to dietary changes and the influence of psychosocial factors such as identity formation, social pressure, and mental health concerns like stress and anxiety [5].

Therefore, interdisciplinary interventions with adolescents can encourage the acquisition of better eating habits that can be maintained throughout adulthood. Furthermore, it is possible to associate behavioral interventions with other educational, social, or environmental strategies, ensuring a better long-term quality of life [6,7]. Interventions targeting adolescent obesity must adopt an interdisciplinary approach, incorporating lifestyle modifications that include dietary changes and increased physical activity practice. Studies indicate that such interventions can significantly reduce body mass index (BMI) and waist circumference [8,9].

However, these interventions must address not only weight loss but also the holistic health of adolescents, encompassing physical, mental, and emotional well-being [10,11,12]. Intervention time can vary between 12 and 24 weeks, with short-term interventions demonstrating improvements in specific settings. However, longer-lasting interventions tend to result in more significant gains, making it easier to maintain the variables verified over time [13]. Long-term interdisciplinary interventions, extending to 24 weeks, have demonstrated more substantial improvements in health-related physical fitness and cardiometabolic risk markers [13,14]. These include enhancements in anthropometry, body composition, muscle strength, endurance, flexibility, and cardiorespiratory fitness, alongside beneficial effects on biochemical markers such as total cholesterol (TC), high-density lipoproteins (HDL-c), low-density lipoproteins (LDL), triglycerides (TG), and fasting glucose [13,14].

The effectiveness of these interventions is often contingent upon sustained engagement from both adolescents and their families, highlighting the need for comprehensive health education to empower adolescents with the knowledge and skills necessary for informed health choices [15,16]. Because of this, the present study aimed to analyze the effects of 12- and 24-week interdisciplinary interventions on health-related physical fitness, biochemical markers, and dietary habits in overweight or obese adolescents. Besides that, the secondary objective was to compare the gender responses between the variables analyzed. As a hypothesis, it was anticipated that extending the intervention duration to 24 weeks would yield more pronounced benefits, including increased lean mass, reduced fat mass, improved physical fitness, and favorable changes in lipid profiles without differences between the genders [15,16]. These interventions were expected to promote healthier dietary patterns, with reduced consumption of processed foods and increased intake of fresh foods, ultimately enhancing adolescents’ overall health. Unlike previous studies that may have focused on specific dietary changes or isolated health outcomes, our research takes a more comprehensive approach by addressing dietary behaviors and their broader impact on physical and mental health, ensuring a more integrated and lasting improvement in adolescents’ well-being.

## 2. Materials and Methods

### 2.1. Experimental Approach to the Problem

This study is a quasi-experimental, longitudinal, and comparative clinical trial. It involved 31 adolescents (final analysis) of both sexes who were classified as overweight or obese. Data collection commenced on 1 March 2022 and concluded on 30 September 2022. The research activities were conducted at the Interdisciplinary Laboratory of Intervention in Health Promotion (LIIPS) facilities at UniCesumar (Maringá, PR, Brazil). The Tool for the Assessment of Study Quality and Reporting in Exercise (TESTEX) scale [17] was used to assess the study’s methodological quality. In case of disagreements, a meeting was held where consensus was established among the researchers.

**Figure 1:**
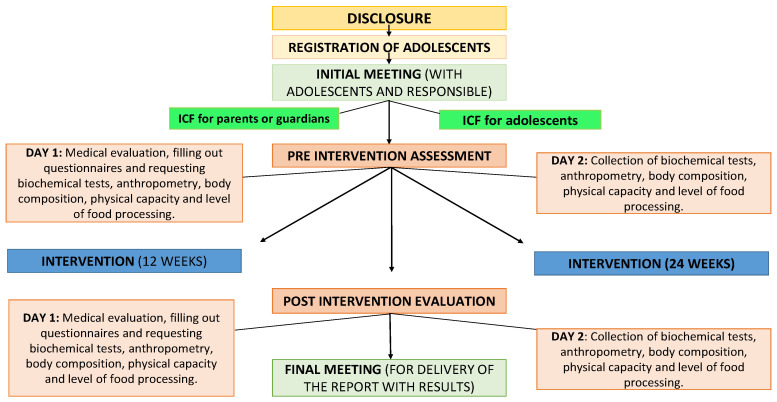
Experimental design of the present study. Note: ICF = informed consent form.

Previously, all parents and guardians of the teenagers were informed about the project’s operation and the study’s purposes. Subsequently, they were invited to sign the free and informed consent form, and the adolescents were also invited to sign it. After signing the terms, the following were scheduled: (I) anamnesis and nutritional clearance (identification of the patient’s history, pre-existing diseases, pubertal stage, level of physical activity, use of medications, screen time, smoking, alcohol consumption, application of questionnaires, among other questions); (II) anthropometry and body composition; collection of blood tests (fasting blood glucose and lipid profile: TC, LDL-c, HDL-c, and TG); and (III) physical fitness tests. Figure 1 presents the experimental design of the research project.

### 2.2. Participants

Seventy adolescents were selected for the study based on the following inclusion criteria: (I) aged between 11 and 17 years; (II) classified as overweight or obese according to BMI; and (III) availability to participate in interdisciplinary interventions twice a week in the evening for 24 weeks. The exclusion criteria included adolescents who: (I) participated in sports activities outside the project; (II) missed more than two consecutive sessions; (III) did not achieve at least 75% attendance in the sessions during the interventions; (IV) failed to complete the required assessments and questionnaires; (V) had orthopedic or cardiovascular issues or cognitive deficits that hindered physical activity; (VI) received nutritional care outside of the research project; and (VII) had medical contraindications to physical activity, as determined through anamnesis, medical consultation, and questionnaire completion.

All participants were informed of the scope of the study and signed an assent and informed consent (parents or legal guardians) form authorizing the use of the information for scientific purposes. The local Research Ethics Committee approved the study through opinion no. 4,913,453/2021. The research project was registered on the Brazilian Clinical Trial Registry Platform (REBEC) under RBR-8fp63gm.

### 2.3. Procedures

The interventions were carried out by professionals duly registered with the council of each profession. Study participants accompanied by their respective legal guardians responded regarding sociodemographic and health characteristics and physical activity practice via the International Physical Activity Questionnaire-IPAQ [18]; (I) if you have already undergone nutritional monitoring at some point; (II) if you usually eat meals in front of the television; (III) history and pre-existing illnesses; and (IV) pubertal staging [19].

### 2.4. Anamnesis and Nutritional Clearance

A professional duly registered with the professional council performed the anamnesis and nutritional diagnosis. The study participants, accompanied by their legal guardians, answered the following questions: (I) age; (II) self-report on race; (III) adolescents’ education; (IV) family income; (V) whether they had health insurance; (VI) whether they used controlled medications; (VII) bowel habits; (VIII) physical activity practice using the International Physical Activity Questionnaire-IPAQ [18]; (IX) whether they had ever had any nutritional monitoring; (X) whether they usually eat meals in front of the television; (XI) history and pre-existing diseases; and (XII) pubertal staging [19].

Pubertal development was monitored using the Tanner Scale [19], which systematized the sequence of pubertal events in both sexes through five stages. This questionnaire was applied in the health promotion laboratory, before and after the interventions, on the first day of assessments, discreetly away from guardians and colleagues so there would be no embarrassment when filling it out. Female participants completed a questionnaire about breast development and the distribution and quantity of hair; male participants completed a questionnaire about the appearance of genitals and the quantity and distribution of pubic hair [20].

### 2.5. Anthropometric and Body Composition Assessment

To assess the morphological aspects of the research participants, height and body weight were recorded, followed by calculation of the BMI (body weight in kilograms divided by height in meters squared), BMI z-score, and body composition using bioelectrical impedance analysis. Height was measured using a stadiometer attached to a digital scale, with an accuracy of 1.0 cm and 100 g (Welmy R-110, Santa Bárbara d’Oeste, São Paulo, Brazil). For body composition analysis, a tetrapolar bioimpedance device (InBody^®^ 570, Biospace Co., Ltd., Seoul, Republic of Korea) was employed, with a capacity of 250 kg and an accuracy of 100 g, following the manufacturer’s instructions and the guidelines of Miller et al. [21], with reliability confirmed by Sergi et al. [22].

### 2.6. Biochemical Tests

Blood collection procedures followed the guidelines of the Clinical and Laboratory Standards Institute. The collected blood samples were distributed in the following tubes: Vacuplast^®^ collection tubes, tubes with the anticoagulant ethylenediamine tetraacetic acid (EDTA) K2, and tubes with the anticoagulant Fluoride/EDTA. Subsequently, to obtain serum and plasma, the samples containing the Fluorine/EDTA activator were centrifuged in a Centrilab^®^ analog centrifuge at 3500 rpm (relative centrifugal force) for 15 min at room temperature [23]. The following laboratory tests were performed: fasting blood glucose and lipid profile (TC, LDL-c, HDL-c, and TG).

### 2.7. Physical Fitness Tests

Research participants were subjected to five physical tests to verify: (I) flexibility of the posterior chain on the right and left sides; (II) maximum isometric handgrip strength of the dominant and non-dominant hands (MIHS); (III) dynamic strength–endurance test for trunk; (IV) isometric general resistance (plank side); and (V) cardiorespiratory fitness. The tests were carried out at baseline, after 12 weeks, and after 24 weeks of interventions by the same evaluators [24].

MIHS was measured using handgrip dynamometer equipment (TKK 5101 Grip-D, Takey, Tokyo, Japan) with the adolescents upright. MIHS was independently measured on both hands (right and left), starting with the dominant side and using standardized procedures with the arm positioned parallel to the trunk. Three measurements were performed for each side, with a 60-s interval between them. The best value for each hand was registered in kilograms force (kgf) [25].

The adolescents’ muscular endurance was measured via the isometric plank strength test [26], elbow flexion and extension test (adapted arm flexion–knees on the floor) [27], and strength–endurance abdominal exercises [28].

When applying the plank strength test, a digital stopwatch from the brand Besportble was used to record the maximum time that the adolescent remained in the prone position, with the hands resting on the ground, with 90° extension of the radioulnar, radiocarpal, and ulnocarpal joints, and 90° flexion of the glenohumeral joints, with a distance of 25 cm from the shoulder line, fingers facing forward, the face aligned with the trunk and legs, supported by the knees or toes on the ground [26].

The elbow flexion and extension test was analyzed based on the number of repetitions the subject could perform. The teenager was positioned with his knees on the floor in the prone position, with arms extended, and he flexed and extended his elbows [27]. The participant was invited to perform dynamic repetitions with full extension and flexion of the elbows, placing the anterior part of the trunk on a mat. Complete repetitions were computed.

The 60-s sit-up test was applied to assess the strength and endurance of the abdominal region [28], recording the number of sit-up repetitions that the adolescent performed during the respective period. The subject was positioned in the supine position on a mat. A sticker was placed on the floor perpendicularly below the hip to demarcate the limit of trunk flexion the adolescent should perform. Complete repetitions were computed during the 60 s of testing [29].

The cardiorespiratory fitness of study participants was assessed using the test of Leger et al. [30], with the equation of Barnett, Chan, and Bruce [31] for adolescents. This test comprises multiple progressive stages of running, with increasing intensity, which determine the subject’s maximum oxygen consumption (VO_2_max). The test begins with a smooth jog (8.5 km/h) between two lines marked on the ground or a court with a minimum width of 20 m. The test ends when the subject cannot keep up with the pace in time or does not reach the marking line 2 consecutive times. VO_2_max was calculated using the following equation = 31.025 + (3.288 × speed in km/h) − (3.248 × age in years) + (0.1536 × age in years × speed in km/h).

### 2.8. Multi-Professional Interventions

Multi-professional interventions were discussed in groups with exercise physiologists, nutritionists, psychologists, medical doctors, and the biomedical team. All activities were developed to stimulate health promotion. The meetings were attended by the nutrition team once a week (lasting 30 min), psychology once a week (30 min), and physical activity twice a week (lasting 60 min).

### 2.9. Nutritional Interventions

The level of food processing was calculated according to the Ministry of Health’s description in the 2014 Food Guide for the Brazilian Population. Based on the information in the guide on fresh, minimally processed, processed, and ultra-processed foods and the classification as less processed and more processed, it was possible to classify the foods consumed by adolescents in calories and grams. The interventions were carried out for 24 weeks, with initial assessments (pre-interventions), after 12 weeks (half of the interventions), and after 24 weeks of interventions (at the end of the interventions).

Nutritional interventions were carried out in groups once a week, with an average duration of 30 min, focusing on changing eating behavior and encouraging healthier eating. Theoretical–practical activities were carried out through expository and dialogue classes, conversation circles, activities in the kitchen of the higher education institution, and other related activities focused on changing eating behavior.

During the 24 weeks of interventions, a structured work schedule was followed, addressing the following topics: initial guidance on the project; food pyramid and preparation of healthy dishes; practical lesson on mindfulness when eating; interpreting labels; physical hunger; emotional hunger; anxiety and eating; building foods, regulatory foods, and energy foods; processed and ultra-processed foods; visual practice of the amount of sugar in processed foods; obesogenic eating and thinking; conversation circle of understanding the topics; behavior change; food on festive days and weekends; dietary fiber; the microbiota and the intestine; conscious eating; diet foods, light foods, and zero foods; and closure of interventions.

### 2.10. Psychoeducation Interventions

Psychoeducation interventions were also carried out in groups once a week, with an average duration of 30 min, aimed at improving adolescents’ mental health with the coordination of a psychologist registered with the regional council of the profession. The activities presented a theoretical-practical approach through conversation circles, debates, outdoor activities, and expository dialogue classes. During the 24 weeks of interventions, a structured work schedule was followed, addressing the following topics: presentation of the themes, schedule, and project objective; what would be normal adolescence and transformations that occur during adolescence; mental transformations in adolescence; social transformations during adolescence; control of emotions; bullying in adolescence; communication in adolescence; anxiety and techniques for controlling anxiety; depression and techniques for dealing with depression; relaxation techniques; full attention to controlling emotions; cell phone use and screen time; communication with parents; acceptance of your daily life; management of emotional problems; nutrition, physical activity, and mental health; the importance of good quality of sleep; and leisure interventions—the playful approach.

### 2.11. Interventions with Physical Activity

The physical activity was designed by exercise physiologists who were not part of the tests before and after the interventions and was carried out at the sports complex. The exercises were conducted in circuit form, emphasizing large muscle groups, with training sessions divided into training plans A and B, with increasing volume and intensity during the intervention period via concurrent training. The intensity of the training sessions was controlled via a rating of perceived exertion (RPE), validated by Foster et al. [32], who indicate a numerical scale from 0 a.u. to 10 a.u., in which 0 represents extremely light effort, and 10 represents extremely maximum effort activity.

With the RPE information and training session time, the internal training load (CTI) was calculated, being RPE x session time in minutes [33]. Hardy and Rejeski’s [34] feelings scale was also applied, which is made up of numbers from −5 (which is equivalent to very bad) to 5 (very good). Participants were asked about their affectivity after the physical activity session. All participants were familiarized with the scales before and during the interventions, receiving explanations previously applied during theoretical classes and before physical activity sessions. The physical training session is presented in Table 1.

**Figure 2:**
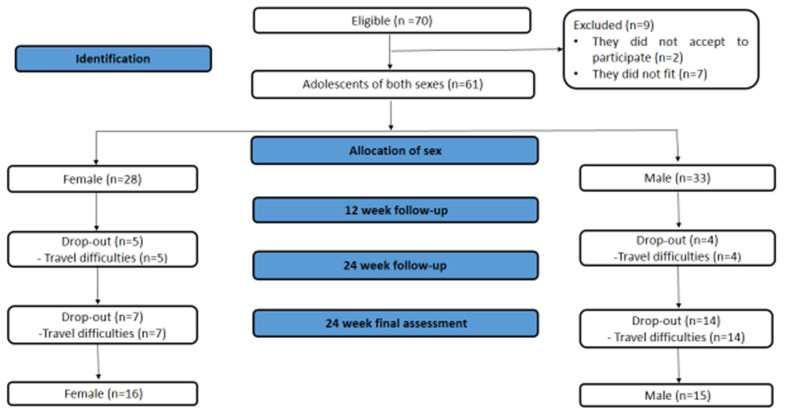
Flowchart of the present study throughout the evaluation process in the 12th and 24th weeks of interventions.

Figure 2 shows the flowchart of the present study. Seventy adolescents were eligible, but 9 were excluded (2 because they did not agree to participate in the research, and 7 did not meet the inclusion criteria). Thus, 61 adolescents of both sexes were divided into two groups (female; n = 28 and male; n = 33). Nine participants withdrew from the interventions after 12 weeks. Twenty-one participants withdrew before the 24-week intervention assessments. Therefore, 16 female and 15 male participants were included in the final assessments. Performing an intention-to-treat analysis was impossible, as participants did not undergo reassessments. However, of the 31 participants who participated, 100% complied with the interventions.

### 2.12. Statistical Analyzes

Statistical analyses were performed using SPSS version 24 software (IBM, Armonk, NY, USA). Data are presented as the mean ± standard deviation (SD). Initially, data normality was tested using the asymmetry-kurtosis test, considering values from 2 to −2 to indicate the need to perform parametric statistical analyses. One-way analysis of variance (one-way ANOVA) was used to locate possible differences between times [35]. The Bonferroni post-hoc test was also used when a significant difference was found [35]. A two-way ANOVA was also applied to compare the effects of interdisciplinary interventions according to gender, applying the Bonferroni post hoc test if a significant difference was found. The significance level established for all tests was *p* < 0.05. The effect size via eta-square (ŋ^2^) was calculated according to the recommendations of Richardson [36]: 0.0099 [small effect], 0.0588 [moderate effect], and 0.1379 [large effect]. Cohen’s d was also calculated for effect size using the following classification: 0.20 [small effect], 0.80 [moderate effect], and >0.80 [large effect] [37].

## 3. Results

Table 2 presents the sociodemographic and health characteristics of the participants in the current study. The average age was 13.74 ± 2.32 years, with a predominance of participants identifying as white. Most families had an income of one to three minimum wages. Participants reported a bowel frequency of two to three times a day. Before the interventions, 51.61% did not engage in physical activity, 45.16% did not receive nutritional support, and 61.29% had lunch while watching television.

No significant differences were observed for IPAQ before, during, and after the interventions (*p* > 0.05). The only difference was the increased physical activity on the days of intervention activities, which was an expected response.

According to the results presented in Table 3, a time effect was observed for FFM (*p* = 0.003; ŋ^2^p = 0.10—moderate effect), with significant increases after 12 and 24 weeks of interventions (*p* < 0.05; for both comparisons). A group effect was observed for FFM in males (*p* = 0.007; ŋ^2^p = 0.08—moderate effect), with higher values after 12 and 24 weeks of interventions (*p* < 0.01; for both comparisons). Likewise, a time effect was verified for LM (*p* = 0.004; ŋ^2^p = 0.09—moderate effect), with the Bonferroni test indicating a significant increase after 24 weeks of interventions (*p* = 0.03). Furthermore, there was a group effect for MM in males (*p* = 0.005; ŋ^2^p = 0.09—moderate effect), with higher values after the 12th week (*p* = 0.01) and 24th week of interventions (*p* = 0.004).

For FM, a time effect was also observed (*p* < 0.001; ŋ^2^p = 0.12—moderate effect), with significant reductions after 12 and 24 weeks of interventions (*p* < 0.01; for both comparisons), and the same was identified for BFP, that is, there was also a time effect (*p* < 0.001; ŋ^2^p = 0.16—large effect), with significant reductions after 12 and 24 weeks of interventions (*p* < 0.01; for both comparisons). Additionally, group effects were observed for FM (*p* = 0.047; ŋ^2^p = 0.07—moderate effect) and BFP (*p* = 0.008; ŋ^2^p = 0.11—moderate), with higher values for the female sex in both comparisons (*p* < 0.01). However, no group, time, or interaction effects were observed for body weight, BMI, SMM, or VF (*p* > 0.05).

Table 4 presents the results of the physical fitness tests carried out pre-interventions, after 12 weeks, and after 24 weeks of interventions. A time effect was observed, with increased flexibility on the right side (*p* < 0.001; ŋ^2^p = 0.15—large effect), with a significant increase after 24 weeks (*p* = 0.04), and on the left side (*p* < 0.001; ŋ^2^p = 0.21—large effect), also with a significant increase after 24 weeks (*p* < 0.001). As a group effect, higher values were detected for the flexibility test on the right side after 12 weeks (*p* = 0.05; ŋ^2^p = 0.08—moderate effect) for males compared to females (*p* = 0.05). Furthermore, there were increases in the number of repetitions of the strength–endurance abdominal test (*p* < 0.001; ŋ^2^p = 0.52—large effect) after 12 weeks (*p* < 0.001) and 24 weeks of interventions (*p* < 0.001).

Concerning the plank test, a significant increase in the length of stay was identified for adolescents (*p* < 0.001 ŋ^2^p = 0.58—large effect), with significant increases after 12 and 24 weeks (*p* < 0.001). However, no significant differences were observed for the MIHS of the dominant and non-dominant hands and the sum of the MIHS across the assessment points performed (*p* > 0.05). Likewise, no group effects were identified nor interactions between MIHS (dominant and non-dominant hands, and sum), flexibility on the left side, abdominal strength–endurance test, elbow flexion and extension, and plank in isometrics (*p* > 0.05). A time effect was identified for VO_2_peak (*p* < 0.001; ŋ^2^p = 0.66—large effect), with higher values after the 12th and 24th weeks of interventions (*p* < 0.001) when compared to pre-intervention values, in addition to higher values in the 24th week when compared to the 12th week, respectively (*p* < 0.001).

The biochemical results in Table 5 showed a time effect for total cholesterol (*p* = 0.014; ŋ^2^p = 0.07—moderate effect), with a significant reduction after 12 weeks (*p* = 0.049). For LDL-c, a time effect was also observed (*p* < 0.001; ŋ^2^p = 0.63—large effect), with significant reductions after 12 and 24 weeks of interventions (*p* < 0.001). A time effect was also observed for HDL-c (*p* < 0.001; ŋ^2^p = 0.73—large effect), with significant increases after 12 and 24 weeks of interventions (*p* < 0.001; for both comparisons). Triglycerides also showed a time effect (*p* < 0.001; ŋ^2^p = 0.16—large effect), with a significant reduction after 24 weeks of interventions (*p* = 0.002). For fasting blood glucose, only a group effect was observed (*p* = 0.003; ŋ^2^p = 0.14– large effect), with higher values for males when compared to females (*p* = 0.003). However, no other differences (group or interaction effects) were observed between total cholesterol, LDL-c, HDL-c, and triglycerides (*p* > 0.05).

**Figure 3:**
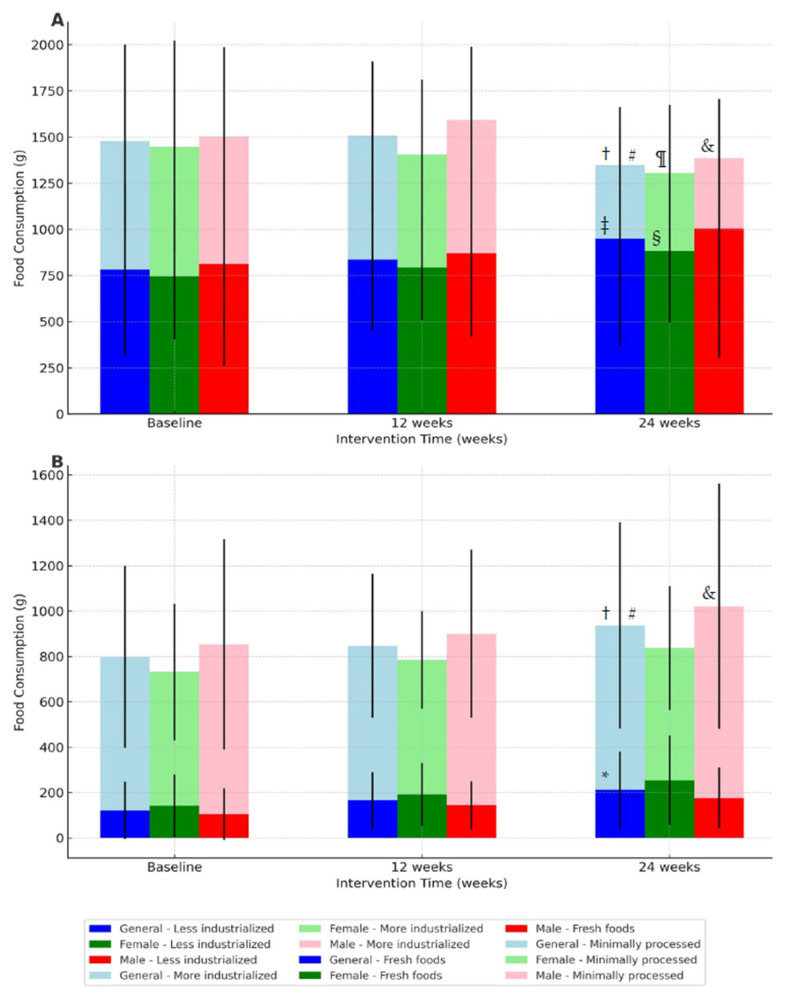
The consumption of less and more processed foods over 24 weeks and food processing level over 24 weeks: (Panel **A**) Consumption of less and more processed foods over 24 weeks; (Panel **B**) Level of food processing over 24 weeks. Note: data are expressed as mean and ± standard deviation; † = significant reduction of more industrialized foods in grams and kilocalories after 24 weeks (*p* < 0.001); ‡ = significant increase of fresh foods in grams after 24 weeks (*p* < 0.001); * = significant increase of fresh foods in kilocalories after 24 weeks (*p* < 0.001); § = significantly higher values for females when compared to males of fresh foods comsuption (*p* = 0.013); & = higher values for males when compared to females consumption of minimally processed foods in grams (*p* = 0.020); with higher values for males when compared to females consumption of minimally processed foods in kilocalories (*p* = 0.01); ¶ = significant reductions after 12 weeks (*p* = 0.002) and 24 weeks of interventions of processed foods in grams (*p* < 0.001); # = significant reduction after 24 weeks for ultra-processed foods in grams (*p* = 0.002).

Figure 3 presents the consumption of less and more processed foods over 24 weeks and the level of food processing over 24 weeks. According to the results observed in the figure above, there was a time effect for the consumption of more industrialized foods in grams (*p* < 0.001; ŋ^2^p = 0.29—large effect) and kilocalories (*p* < 0.001; ŋ^2^p = 0.29—large effect), with a significant reduction after 24 weeks of interventions (*p* < 0.001). However, no significant differences were observed in the consumption of less processed foods (*p* > 0.05), nor were there any differences between groups or interactions (*p* > 0.05).

The data presented in Figure 3 show the level of food processing over 24 weeks of interventions. A time effect was identified for the consumption of fresh foods in grams (*p* < 0.001; ŋ^2^p = 0.19—large effect), with a significant increase after 24 weeks of interventions (*p* = 0.001). Likewise, there was a time effect for the consumption of fresh foods in kilocalories (*p* < 0.001; ŋ^2^p = 0.21—large effect), with an increase after 24 weeks of interventions (*p* < 0.001). Furthermore, there was a group effect for the consumption of fresh foods (*p* = 0.013; ŋ^2^p = 0.10—moderate effect), with significantly higher values for females when compared to males (*p* = 0.013). A group effect was also identified for the consumption of minimally processed foods in grams (*p* = 0.020; ŋ^2^p = 0.09—moderate effect), with the Bonferroni test indicating higher values for males when compared to females (*p* = 0.02). Furthermore, a group effect was observed for the consumption of minimally processed foods in kilocalories (*p* = 0.019; ŋ^2^p = 0.09—moderate effect), with higher values for males when compared to females (*p* = 0.01).

Considering processed foods in grams, a time effect was identified (*p* = 0.002; ŋ^2^p = 0.10—moderate effect), with significant reductions after 12 weeks (*p* = 0.002) and 24 weeks of interventions (*p* < 0.001). A time effect was identified for ultra-processed foods in grams (*p* = 0.002; ŋ^2^p = 0.10—moderate effect), with a significant reduction after 24 weeks of interventions (*p* = 0.027). Likewise, a time effect was also identified for the consumption of ultra-processed foods in kilocalories (*p* < 0.001; ŋ^2^p = 0.20—large effect), with significant reductions after 12 weeks (*p* = 0.01) and 24 weeks of interventions (*p* < 0.001). However, no time effects were observed for consuming minimally processed foods over the 24 weeks of interventions (*p* > 0.05). Finally, there were no other group effects or interactions for the other variables related to the level of food processing (*p* > 0.05). Figure 3 presents the consumption of less and more processed foods over 24 weeks and the food processing level over 24 weeks.

**Figure 4:**
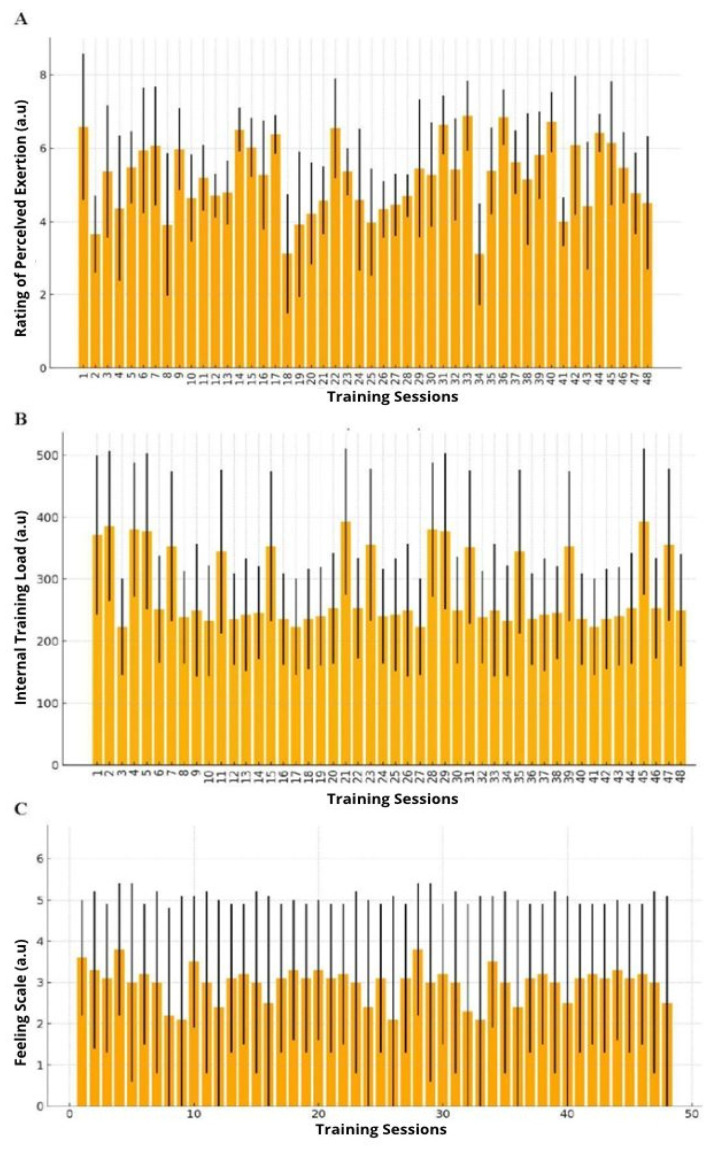
Ratings of perceived exertion, internal training load, and feeling scale over 48 training sessions: (Panel **A**) Rating of perceived exertion; (Panel **B**) Internal training load; and (Panel **C**) Feeling scale of groups throughout 48 training sessions. Note: data are expressed as mean and ± standard deviation; a.u., arbitrary unit.

Figure 4 shows the perceived exertion, internal training load, and feeling scale ratings over 48 training sessions. Significant differences were observed for internal training load over the 48 physical activity sessions. However, such responses were also suppressed to synthesize this information, which is not the central focus of the research. Significant differences were observed in the rating of perceived exertion throughout the 48 physical activity sessions. However, such responses were suppressed to synthesize this information, which is not the central focus of the research. The figure below shows the internal training load of the adolescents participating in the research. No significant differences were observed in the participants’ affective responses in the present study during the interdisciplinary interventions (*p* > 0.05). The figure below shows the participants’ ratings of perceived exertion over the 24 weeks of interventions.

## 4. Discussion

This study aimed to investigate the effects of 12 and 24 weeks of interdisciplinary interventions on health-related physical fitness, biochemical markers, and level of food processing in overweight or obese adolescents. Therefore, the main outcomes of the present study indicated: (I) increases in FFM and LM after 12 and 24 weeks of interventions for males and females; (II) reductions in absolute and relative FM after 12 and 24 weeks of interventions; (III) increases in flexibility on the right and left sides for males and females after 24 weeks of interventions; (IV) higher values for flexibility in males when compared to females; (V) increases in the number of repetitions in elbow and abdominal flexions and extensions, as well as increases in the time spent in isometric plank after 12 and 24 weeks of interventions; (VI) a reduction in total cholesterol after 12 weeks and a reduction in triglycerides after 24 weeks of interventions; (VII) reductions in LDL-c after 12 and 24 weeks of interventions and an increase in HDL-c after 24 weeks of interventions; (VIII) a reduction in the consumption of more processed foods after 24 weeks of interventions; (IX) an increase in the consumption of fresh foods in grams and kilocalories; (X) higher values for the consumption of fresh foods in females when compared to males and higher values for the consumption of minimally processed foods in males when compared to females; (XI) reductions in the consumption of processed and ultra-processed foods after 12 and 24 weeks of interventions. Based on the results, the study hypothesis was confirmed.

It is already well established in the literature that interdisciplinary interventions promote anthropometric, body composition, and nutritional improvements [10,11,12,29,34]. Therefore, it is possible to state that the interdisciplinary model proposed with theoretical-practical nutrition classes, theoretical psychoeducation classes, and physical education practice improved the physical health of the adolescents participating in the study, regardless of sex. Therefore, it is considered that interdisciplinary interventions can be carried out jointly between pre-adolescents and adolescents of both sexes [3] and can be expanded to population interventions—that is, they are low-cost. Since prevention is significantly less expensive than treatment for governmental and non-governmental organizations, managers can effectively direct care and health promotion initiatives for the population [34].

Krebs et al. [38] point out that nutritional interventions (diets rich in protein and low in carbohydrates vs. diets low in lipids) and encouraging daily physical activity practice (around 30 min/day of moderate to vigorous intensity) promoted losses of significant weight after 12 and 24 weeks of interventions. Vermeiren et al. [39] discussed that dietary interventions can lead to weight regain, although research in this direction is still in its infancy. However, according to the same authors, the cardiometabolic benefits of weight loss were maintained, even after regaining weight, and the prevalence of metabolic syndrome (a combination of 3 or more cardiometabolic risk factors) tended to decrease temporarily. However, care must be taken; metabolically healthy obesity cannot be considered safe, although it can direct individual actions to manage the disease [40]. It is also worth noting that around 55% of children who are obese in childhood become obese in adolescence, and 80% of obese adolescents will remain obese in adulthood [41].

Body weight and BMI did not show significant differences at the two assessment points, which aligned with the study by Gillette and collaborators [13]. Therefore, it is discussed in the literature that BMI is not a good indicator for evaluating possible changes induced by physical activity and nutritional guidance [10,11,12]. This is due to the exchange of body composition components, such as the breakdown and oxidation of FM and the hypertrophy of skeletal striated muscle. Therefore, if the exchange of body composition components is “proportional”, there will be no body weight and BMI changes. However, a previous study that carried out interdisciplinary interventions to increase physical activity and food consumption of adolescents with severe obesity identified a significant reduction in BMI after 24 weeks of interventions [8]. Thus, the analysis of BMI for adolescents with severe obesity can be a supplementary tool for analyzing body composition, that is, anthropometry and body composition.

FFM was significantly increased after 12 and 24 weeks compared to pre-intervention values. Additionally, FFM values in male adolescents were significantly higher than in females—these results can be explained by the morphophysiological differences of both sexes [42]. However, it is believed that the stabilization of FFM between the 12th and 24th week is related to a stabilization of the volume and intensity of the physical efforts performed (according to data on the subjective perception of effort and internal training load), in addition to the adaptations generated throughout the first 12 weeks of interventions that are related to significant gains, while more trained individuals require greater training sessions for muscular hypertrophy [43]. FM and BF showed significant reductions after the 12th and 24th weeks of intervention.

The reduction until the 12th week corroborates previous studies with a similar experimental design [10,11,12]. On the other hand, the absence of differences between interventions focused on weight loss in adolescents with obesity between points after 12 and 24 weeks of interventions seems to be related to a “plateau effect” [8].

A group effect was also observed, with significantly higher values for FM and BFP in females than males. Due to morphophysiological issues (body composition and hormonal aspects), female adolescents have greater adipose mass than males, and this response was already expected [44]. The literature has discussed the issue related to the “plateau effect”, with it being hypothesized that the stabilization of weight loss is related to partial adherence to interventions to change behavior, especially about diet, and not a metabolic adaptation [45].

However, it has been argued that each kg of body mass lost tends to increase appetite by ~100 kcal/day, corresponding to a 3× better number of adaptations generated by the energy expenditure caused [46]. It is worth noting that the participants in the present study did not follow a nutritional plan; they carried out interventions focusing on nutritional education and psychoeducation, the second mainly aimed at managing stress, anxiety, depression, and binge eating, aspects that are associated with excess weight and obesity [47]. Considering the impossibility of carrying out an “effectiveness trial”, that is, controlled responses within a model considered ideal, new studies seek to investigate “effectiveness”, that is, whether an intervention has an effect in the real world [48]. Therefore, new trials that test different intervention models for the management of obesity in adolescents may help health professionals and managers to outline strategies to be applied in health services [48].

No significant differences were observed at the different assessment points for MIHS. Considering that no isometric or maximal strength exercises were performed, the absence of differences over the 24 weeks can be justified [12]. Flexibility increased significantly after 24 weeks of interventions. Donti et al. [49] discuss that flexibility gains are related to the volume and intensity intended to work on this component of physical fitness. In this sense, Panidi et al. [50] found an increase in flexibility after 12 weeks of high-volume interventions, which were accompanied by an increase in the length of the medial gastrocnemius fascicle, larger stretching of the medial and lateral gastrocnemius, in addition to an increase in the cross-sectional area of the gastrocnemius in adolescent athletes.

However, one of the differences between the study by Panidi et al. [50] and the present study refers to the protocol used, that is, while the 12-week research carried out exercises focused on increasing flexibility, our study used a multicomponent approach. Our study divided the time between muscle strength and endurance activities, cardiorespiratory fitness, and flexibility twice a week for 60 min. Thus, the stimuli aimed at flexibility in the present study are related to low-intensity actions, justifying the improvement of this component of physical fitness after 24 weeks of interventions.

Significant improvements were observed after 12 and 24 weeks of interventions for flexion and extension of elbows, sit-ups, and plank in isometrics, tests related to dynamic (first 2) and isometric (last) muscular resistance. Previous scientific evidence has shown similar responses with improved muscular resistance after 12 weeks of interventions [10,11,12,50]. The VO_2_ peak improved significantly after 12 and 24 weeks of interventions and presented significantly higher values after the 24th week of interventions when compared to the 12th. In this aspect, increasing cardiorespiratory fitness reflected a positive response to the interventions since improving cardiorespiratory fitness reduces the risks associated with morbidity and mortality and is considered a key component of cardiovascular health in adolescents [10,11,12].

Improvements in different components of physical fitness do not occur linearly and may not occur in the same period. In this sense, Reyes-Laredo et al. [51] pointed out that the physical capabilities of athletes may present different response times depending on the stimuli performed. Consequently, improving physical fitness components, such as muscle strength and endurance, flexibility, and cardiorespiratory fitness, tend to respond individually (less volume, intensity, and frequency or greater volume, intensity, and frequency of the stimuli performed).

Total cholesterol and TG were significantly reduced after 24 weeks of interventions, LDL-c, and fasting blood glucose were significantly reduced after 12 and 24 weeks of interventions, and HDL-c increased significantly after 12 and 24 weeks. Similar responses were identified by Staiano et al. [52] after 24 weeks of home interventions with exergames associated with telehealth monitoring (interdisciplinary actions) in overweight or obese adolescents. Given this, Vasconcellos et al. [53] pointed out that physical activity directly reduces cardiometabolic risk (better glycemic control, reduced triglycerides, total cholesterol, and LDL-c) and improves adolescent body composition. The scientific literature is already very consistent regarding the improvement of cardiometabolic risk parameters after interdisciplinary interventions in overweight or obese adolescents [10,11,12,29].

There was also an improvement in the glycemic control of adolescents, especially males who had “pre-diabetes” [54], but after the interventions, these values were reduced to “borderline”, indicating a significant reduction in fasting blood glucose. Considering the different periods (12 or 24 weeks of intervention) to improve the different cardiometabolic risk parameters, it is believed that the most relevant point is maintaining a healthy and active lifestyle to provide higher health and quality of life for teenagers. The findings reinforce the need for prolonged interventions to improve the biopsychosocial health of adolescents [15,55,56].

It is also discussed that parents can influence their children’s food choices [55], although, in some circumstances, this influence does not seem to differ when parents and/or guardians participate in interventions focused on improving quality diet, cardiometabolic risk, and anthropometry/969 body composition of overweight or obese adolescents [57]. Therefore, it is conjectured that interdisciplinary interventions to improve the biopsychosocial aspects of overweight or obese adolescents should be based on evidence-based practice, in addition to directing efforts toward personalized interventions, if possible [58].

On the other hand, if it is impossible to promote personalized actions, it is considered that interventions can focus on nutritional education, psychoeducation, and physical activity in homogeneous groups, i.e., participants with the same characteristics can share their desires, difficulties, barriers, and even improvements throughout interdisciplinary interventions, since there are no single protocols that suit all people seeking assistance [59].

It is essential to highlight that adolescence represents a crucial time for establishing the foundations of health in adult life. During this phase of biological and social transformations, it is common for adopted dietary patterns to become less healthy, requiring specific intervention approaches targeted at this age group [15]. Given this, health education is an essential strategy to improve adolescents’ knowledge, attitudes, and practices concerning nutrition and physical activity in a way that incorporates health promotion practices based on established guidelines and supported by policies that promote health education [15]. In this way, the level of health knowledge among adolescents will be increased to prevent or minimize health problems and encourage healthy practices. Understanding and utilizing health information is central to young people making informed choices to improve their well-being, mainly through effective communication about health issues [60].

Therefore, in the present study, it was possible to observe that after 24 weeks of interventions, there were decreases in the consumption of industrialized, processed, and ultra-processed foods in grams and calories, as well as an increase in the consumption of fresh foods in grams and calories, which may be associated with promoting and improving health, thus contributing to calorie loss, as observed, as they are foods that tend to be healthier and have lower energy density [29].

Regarding the increased consumption of fresh and minimally processed foods and the decreased intake of processed and ultra-processed products, theoretical-practical interventions improved the quality of adolescents’ diets. Another point observed was that the consumption of fresh foods among females was significantly higher than that among males, and the consumption of minimally processed foods was higher among males than females. In this sense, the responses suggest that male adolescents choose healthier foods that are ready to eat, such as minimally processed foods.

These interventions are believed to promote healthy autonomy in food choices and the adoption of healthy behaviors to prevent adolescent obesity [41,61,62]. In addition, this study found better results with 24 weeks of interventions in body composition and dietary profile practices, suggesting better results than 12-week interventions guided by the American Guide to Obesity Management [63].

The results of the present study indicate positive responses to physical activity sessions over the 48 sessions. Positive responses to the physical activity performed were stable during the interventions (~3 points, indicating “good” responses). It was found that RPE varied between stimuli 4 and 7 (moderate to intense efforts), although the efforts were mostly moderate. Branco et al. [12], in 12 weeks of interdisciplinary interventions with overweight or obese adolescents, found that the internal training load ranged between ~200 a.u. to 400 a.u. until the 24th training session and ~600 a.u. from the 25th to 28th session, respectively. When analyzing the result of the internal training load, it is inferred that the adolescents adapted to the intensity of the stimuli performed despite the researchers manipulating the volume and intensity of efforts during the interventions. It is believed that the process of continuing education to fill in the information on RPE in conjunction with the goals of the sessions established and constant feedback of information can more assertively guide the expectations of the professionals linked to physical activity who conduct the sessions together with the adolescents participating in the study.

The limitations of the present study can be verified through the methodological quality of the study by TESTEX, where it obtained 10/15 points on the scale, pointing out that there were limitations in criteria 2 and 3 of the scale, referring to randomization and allocation concealment, respectively, which were not met. Criterion 4 of the scale was considered one point even with the morpho-physical differences between the sexes of the girl groups and the boy groups. Regarding criterion 7, it was impossible to conduct an intention-to-treat analysis as study participants who dropped out during the intervention weeks did not return for reassessments. Moreover, criterion 10 was not included because there was no control group.

## 5. Conclusions

The interdisciplinary interventions showed benefits after 12 and 24 weeks of interventions in the adolescents participating in the present study. The 12-week interventions showed satisfactory results but not in all the biochemical and nutritional parameters analyzed. On the other hand, the continuity of interventions lasting 24 weeks promoted better outcomes for biochemical variables, level of food processing (with an increase in the consumption of fresh foods and reductions in the consumption of processed and ultra-processed foods), in addition to a significant reduction in the consumption of processed foods.

Adolescents of both sexes generally responded positively to the interventions since practically all the variables analyzed showed similar results after 12 and 24 weeks of interventions. Given this, it is considered that the intervention model can be applied to both sexes. Another relevant point concerns the affective responses that remained positive and constant throughout the 24 weeks. This intervention model had playful characteristics, such as using ropes, balls, and other tools within the training itself with body weight and accessories, in addition to the motivational dynamics provided by the physical education professionals who conducted the physical activity sessions.

Finally, more prolonged interventions to improve the physical, metabolic, and nutritional health of overweight or obese adolescents are recommended to provide positive adjustments in the empowerment process of health promotion in the biopsychosocial sphere, with the proposed intervention program being advantageous, as can be seen in the results, with a reduction in obesity, through health education and awareness.

## Figures and Tables

**Table 1:** Systematization of concurrent training plans A and B.

Training Plan A					
Order	Exercises	Series	Repetition(s)	Rest(s)	Cadency
1	Warm up—walking, interval running	10′	-	-	-
2	Step (goes up and down)	3x	30″ or 40″	30″ or 20″	1:1
3	Rope tsunami	3x	30″ or 40″	30″ or 20″	1:1
4	Row with squat	3x	30″ or 40″	30″ or 20″	1:1
5	Jumps	3x	30″ or 40″	30″ or 20″	1:1
6	Elevation of pelvis	3x	30″ or 40″	30″ or 20″	1:1
7	Frontal displacement (cone with a jump at the end)	3x	30″ or 40″	30″ or 20″	1:1
8	Squat with simultaneous curl	3x	30″ or 40″	30″ or 20″	1:1
9	Ladder agility (front)	3x	30″ or 40″	30″ or 20″	1:1
10	Supra-abdominal with adduction	3x	30″ or 40″	30″ or 20″	1:1
11	Shifting with mini band	3x	30″ or 40″	30″ or 20″	1:1
12	Active cooldown	2′	30″ or 40″	30″ or 20″	1:1
**Training Plan B**					
1	Warm up—walking, interval running	10′	-	-	-
2	Step lateral (goes up and down)	3x	30″ or 40″	30″ or 20″	1:1
3	Elastic traction	3x	30″ or 40″	30″ or 20″	1:1
4	Arm flexion	3x	30″ or 40″	30″ or 20″	1:1
5	Squat with ball throw down	3x	30″ or 40″	30″ or 20″	1:1
6	Agility ladder (side)	3x	30″ or 40″	30″ or 20″	1:1
7	Infra-abdominal (feet holding the ball)	3x	30″ or 40″	30″ or 20″	1:1
8	Squat with development	3x	30″ or 40″	30″ or 20″	1:1
9	Zig-zag shift on the disk	3x	30″ or 40″	30″ or 20″	1:1
10	Jump	3x	30″ or 40″	30″ or 20″	1:1
11	Adapted burpee	3x	30″ or 40″	30″ or 20″	1:1
12	Active cooldown	2′	***	***	1:1

**Table 2:** Sociodemographic and health characteristics of the participants in the present study.

Variables	N (%)
Sex	
Female	16 (51.61)
Male	15 (48.38)
Age	13.74 ± 2.32
Race	
White	23 (74.19)
Brown	7 (22.58)
Black	1 (3.22)
School level	
Elementary school	30 (96.78)
High school	1 (3.22)
Family income	
1 to 2 minimum wages	15 (48.38)
2 to 3 minimum wages	15 (48.38)
Above 3 minimum wages	1 (3.22)
Has a health plan	
Yes	10 (32.25)
No	21 (67.74)
Medicine	
Antidepressant	3 (9.67)
Mood stabilizer	2 (4.45)
Thyroid hormone	2 (6.45)
None	24 (67.74)
Intestinal habit	
Frequency 1 to 2 times a day	8 (25.80)
Frequency 2 to 3 times a day	14 (45.16)
Up to 3 days without having a bowel movement	5 (16.12)
More than 3 days without having a bowel movement	4 (12.90)
Practice of physical activity (before the interventions)	
Yes	15 (48.38)
No	16 (51.61)
Frequency per week	
1 turn	4 (26.66)
2 to 3 times	6 (19.35)
4 to 5 times	4 (26.66)
7 times	1 (3.22)
Activity duration	
Up to 30 min	3 (9.67)
1 to 2 h	8 (25.80)
2 h	2 (6.45)
More than 2 h	2 (6.45)
0	15 (48.38)
Screen time	
3 to 10 h	10 (3.22)
10 to 16 h	6 (19.35)
0	15 (48.38)
Have you already undergone nutritional support	
Yes	17 (54.83)
No	14 (45.16)
Meals in front of the television	
Yes	19 (61.29)
No	12 (38.70)
Pubertal development—Tanner	
Female	Pre: 4.0 ± 0.4; post 12 week: 4.3 ± 0.5 *; and post 24 week: 4.3 ± 0.5 *
Male	Pre: 3.5 ± 1.3; post 12 week: 3.6 ± 1.2; and post 24 week: 3.7 ± 1.1

Note: data are expressed as absolute and relative frequency as well as mean and standard deviation (±); * = timing effect with significant difference between the weeks.

**Table 3:** Anthropometric and body composition parameters during the intervention process in male, female, and general (together) adolescents.

Anthropometry and Body Composition		Baseline	12 Weeks	Cohen’s d (12 Weeks and Baseline)	24 Weeks	Cohen’s d (24 Weeks and Baseline)
Body mass (kg)	G	79.5 ± 26.8	79.3 ± 25.4	0.00—small effect	78.1 ± 26.8	−0.05—small effect
	F	83.5 ± 29.9	82.5 ± 28.0	−0.03—small effect	78.7 ± 29.8	−0.16—small effect
	M	76.1 ± 23.8	76.5 ± 23.0	0.01—small effect	77.5 ± 24.5	0.05—small effect
Height (m)	G	1.63 ± 0.08	1.63 ± 0.08	0.00—small effect	1.64 ± 0.08	0.12—small effect
	F	1.60 ± 0.05	1.60 ± 0.05	0.00—small effect	1.61 ± 0.05	0.20—small effect
	M	1.67 ± 0.10	1.67 ± 0.10	0.00—small effect	1.67 ± 0.10	0.00—small effect
BMI (kg/m^2^)	G	29.9 ± 8.4	29.7 ± 7.9	−0.02—small effect	29.2 ± 8.5	−0.08—small effect
	F	32.0 ± 9.7	31.6 ± 8.9	−0.04—small effect	31.3 ± 8.5	−0.07—small effect
	M	28.2 ± 6.9	28.2 ± 6.7	0.00—small effect	27.4 ± 8.3	−0.11—small effect
BMI z-score	G	1.85 ± 0.73	1.92 ± 0.65	0.10—small effect	1.90 ± 0.65	−0.07—small effect
	F	2.09 ± 0.75	2.09 ± 0.71	0.00—small effect	2.09 ± 0.67	0.00—small effect
	M	1.56 ± 0.62	1.72 ± 0.56	0.27—moderate effect	1.67 ± 0.58	0.18—small effect
FFM (kg) ‡†	G	45.8 ± 11.0	46.4 ± 10.8	0.05—small effect	46.7 ± 10.6	0.08—small effect
	F	44.7 ± 10.3	44.8 ± 9.9	0.00—small effect	44.8 ± 9.4	0.00—small effect
	M	46.8 ± 11.6	47.8 ± 11.5	0.08—small effect	48.3 ± 11.4	0.12—small effect
LM (kg) ‡†	G	43.2 ± 10.4	43.7 ± 10.2	0.04—small effect	43.9 ± 10.0	0.06—small effect
	F	42.1 ± 9.8	42.2 ± 9.4	−0.01—small effect	42.1 ± 8.9	0.00—small effect
	M	44.0 ± 10.9	45.0 ± 10.8	0.09—small effect	45.5 ± 10.7	0.13—small effect
SMM (kg)	G	25.2 ± 6.5	25.4 ± 6.4	0.03—small effect	26.3 ± 7.0	0.17—small effect
	F	24.4 ± 6.1	24.4 ± 5.8	0,00—small effect	24.4 ± 5.5	0.00—small effect
	M	25.9 ± 6.8	26.3 ± 6.9	0.05—small effect	27.9 ± 7.8	0.29—moderate effect
FM (kg) ‡§	G	33.7 ± 18.5	32.9 ± 17.6	−0.04—small effect	32.4 ± 17.4	−0.07—small effect
	F	38.8 ± 20.3	37.7 ± 19.0	−0.05—small effect	37.1 ± 18.1	−0.08—small effect
	M	29.4 ± 15.9	28.8 ± 15.5	−0.03—small effect	28.3 ± 16.0	−0.06—small effect
BFP (%) ‡§	G	39.9 ± 10.9	39.1 ± 11.0	−0.07—small effect	38.6 ± 11.6	−0.10—small effect
	F	43.7 ± 9.0	43.1 ± 9.0	−0.06—small effect	42.9 ± 8.9	−0.08—small effect
	M	36.7 ± 11.5	35.7 ± 11.5	−0.08—small effect	34.9 ± 12.5	−0.15—small effect

Note: data are expressed as mean and standard deviation (±). G = general; F = female; M = male; BMI = body mass index; FFM = fat-free mass; LM = lean mass; MME = skeletal muscle mass; FM = fat mass; BFP = body fat percentage; ‡ = timing effect (pre-interventions vs. 12 weeks and 24 weeks of interventions; *p* < 0.05); § = group effect (*p* < 0.05, male vs. female); † = interaction between time and group (*p* < 0.05).

**Table 4:** Physical fitness parameters during the intervention process in male, female, and general (together) adolescents.

Health-Related Physical Fitness Parameters		Baseline	12 Weeks	Cohen’s d (12 Weeks and Baseline)	24 Weeks	Cohen’s d (24 Weeks and Baseline)
MIHS dominant hand (kgf)	G	22.9 ± 8.5	23.0 ± 8.5	0.01—small effect	23.0 ± 8.3	0.01—small effect
	F	22.5 ± 8.9	22.4 ± 8.7	−0.01—small effect	22.2 ± 8.3	−0.03—small effect
	M	23.2 ± 8.3	22.5 ± 8.5	−0.08—small effect	23.6 ± 8.4	0.04—small effect
MIHS non dominant hand (kgf)	G	20.9 ± 8.5	20.8 ± 8.1	−0.01—small effect	20.5 ± 8.3	−0.04—small effect
	F	20.0 ± 8.8	20.2 ± 8.2	0.02—small effect	19.7 ± 8.5	−0.03—small effect
	M	21.7 ± 8.4	21.3 ± 8.1	−0.00—small effect	21.1 ± 8.2	−0.07—small effect
SMIHS (kgf)	G	43.7 ± 16.7	43.8 ± 16.3	0.00—small effect	43.4 ± 16.3	−0.01—small effect
	F	42.3 ± 17.2	42.6 ± 16.6	0.01—small effect	41.9 ± 16.6	−0.02—small effect
	M	45.0 ± 16.4	44.8 ± 16.2	−0.01—small effect	44.7 ± 16.2	−0.01—small effect
Flexibility—R (cm) ¶†	G	25.8 ± 7.5	26.0 ± 7.4	0.02—small effect	27.1 ± 8.1	0.17—small effect
	F	24.3 ± 7.1	25.1 ± 6.5	0.01—small effect	26.7 ± 7.5	0.33—moderate effect
	M	27.1 ± 7.8	26.8 ± 8.1	−0.03—small effect	27.4 ± 8.6	0.03—small effect
Flexibility—L (cm) ¶	G	25.6 ± 7.5	25.9 ± 7.6	0.03—small effect	27.5 ± 7.4	0.25—moderate effect
	F	24.4 ± 6.6	24.8 ± 6.2	0.06—small effect	27.0 ± 6.0	0.39—moderate effect
	M	26.5 ± 8.1	26.9 ± 8.7	0.04—small effect	27.9 ± 8.5	0.17—small effect
Flexion and extension of elbows (reps/min) ‡	G	10.5 ± 8.5	13.8 ± 8.3	0.39—moderate effect	17.6 ± 7.1	0.83—large effect
	F	9.5 ± 8.2	12.9 ± 7.8	0.42—moderate effect	16.7 ± 7.0	0.87—large effect
	M	11.4 ± 8.8	14.6 ± 8.7	0.36—moderate effect	18.4 ± 7.1	0.79—moderate effect
Strength–endurance abs (reps/min) ‡	G	24.6 ± 8.5	27.1 ± 8.7	0.29—moderate effect	30.4 ± 8.4	0.68—moderate effect
	F	25.8 ± 8.2	28.3 ± 8.5	0.29—moderate effect	32.0 ± 7.9	0.75—moderate effect
	M	23.6 ± 8.8	26.0 ± 8.9	0.27—moderate effect	29.0 ± 8.7	0.61—moderate effect
Plank torso in isometrics (s) ‡	G	39.6 ± 27.1	45.6 ± 22.7	0.24—moderate effect	55.1 ± 23.3	0.57—moderate effect
	F	42.1 ± 29.6	47.9 ± 25.9	0.20—small effect	58.3 ± 26.3	0.54—moderate effect
	M	37.5 ± 25.1	43.6 ± 19.7	0.27—moderate effect	52.4 ± 20.5	0.59—moderate effect
VO_2_peak (mL/kg/min) &¶	G	23.67 ± 5.22	25.48 ± 4.74	0.36—moderate effect	26.90 ± 4.54	0.61—moderate effect
	F	22.64 ± 4.97	24.67 ± 4.25	0.43—moderate effect	25.93 ± 3.76	0.66—moderate effect
	M	24.64 ± 5.43	26.24 ± 5.18	0.30—moderate effect	27.81 ± 5.12	0.58—moderate effect

Note: data are expressed as mean and standard deviation (±). G = general; F = female; M = male; MIHS dominant hand = maximum isometric handgrip strength of dominant hand; MIHS non-dominant hand = maximum isometric left handgrip strength of non-dominant hand; SMIHS = sum of maximum isometric handgrip strength (dominant and non-dominant hand); & = timing effect (pre-interventions vs. after 12 weeks of interventions, *p* < 0.05); ¶ = timing effect (pre-interventions vs. after 24 weeks of interventions, *p* < 0.05); ‡ = timing effect (pre-interventions vs. 12 weeks and 24 weeks of interventions, *p* < 0.05); † = interaction between time and group (*p* < 0.05).

**Table 5:** Biochemical parameters during the intervention process in male, female, and general (together) adolescents.

Biochemical Parameters		Baseline	12 Weeks	Cohen’s d (12 Weeks and Baseline)	24 Weeks	Cohen’s d (24 Weeks and Baseline)
TC (mg/dL) *	G	172.3 ± 27.7	168.0 ± 27.2	−0.15—small effect	164.9 ± 26.7	−0.27—moderate effect
	F	169.4 ± 28.	168.6 ± 29.0	−0.02—small effect	167.5 ± 25.2	−0.06—small effect
	M	174.8 ± 26.8	167.6 ± 26.0	−0.27—moderate effect	167.7 ± 28.1	0.26—moderate effect
LDL-c (mg/dL) ‡	G	132.4 ± 25.1	111.2 ± 23.1	−0.87—large effect	99.8 ± 22.8	−1.29—large effect
	F	129.2 ± 26.2	110.0 ± 25.1	−0.74—moderate effect	100.0 ± 24.2	−1.11—large effect
	M	132.0 ± 24.1	112.2 ± 21.6	−0.86—large effect	99.6 ± 22.0	−1.34—large effect
HDL-c (mg/dL) ‡	G	37.6 ± 3.5	42.5 ± 12.7	0.52—moderate effect	49.1 ± 12.2	3.3—large effect
	F	36.9 ± 3.8	43.5 ± 11.9	0.74—moderate effect	51.3 ± 11.8	3.8—large effect
	M	38.2 ± 3.2	41.6 ± 13.5	0.34—moderate effect	47.3 ± 12.4	2.8—large effect
TG (mg/dL) *	G	77.8 ± 23.9	77.2 ± 25.5	−0.02—small effect	72.6 ± 25.5	0.21—moderate effect
	F	83.8 ± 25.6	83.5 ± 27.9	−0.01—small effect	76.6 ± 26.6	−0.28—moderate effect
	M	72.8 ± 21.4	71.8 ± 22.3	−0.06—small effect	69.2 ± 24.5	−0.17—small effect
Fasting glucose (mg/dL) ‡§	G	106.2 ± 9.6	102.8 ± 8.4	−0.20—small effect	97.0 ± 9.4	−0.95—large effect
	F	102.3 ± 7.3	99.9 ± 5.7	−0.36—moderate effect	93.5 ± 7.3	−1.20—large effect
	M	109.5 ± 10.2	105.2 ± 9.5	−0.43—moderate effect	100.0 ± 10.1	−0.93—large effect

Note: data are expressed as mean and standard deviation (±). G = general; F = female; M = male; TC = total cholesterol; LDL-c = low-density lipoprotein; HDL-c = high-density lipoprotein; TG = triglycerides. * = timing effect (pre-interventions vs. 24 weeks of interventions; *p* < 0.05); ‡ = timing effect (pre-interventions vs. 12 and 24 weeks of interventions; *p* < 0.05); § = group effect (male vs. female; *p* <0.05).

## Data Availability

All data is available upon request.
